# Development of and Testing Novel Questionnaires Assessing Palliative Care-Related Knowledge, Attitudes, and Confidence Among Home Healthcare Clinicians, Patients, and Caregivers

**DOI:** 10.1097/NHH.0000000000001316

**Published:** 2025-01-07

**Authors:** Ashley M. Chastain, Jingjing Shang, Komal P. Murali, Lori King, Charity Ogunlusi, Suning Zhao, Jung A. Kang, Yihong Zhao, Khadra Dualeh, Margaret V. McDonald

**Affiliations:** **Ashley M. Chastain, DrPH, MPH**, is the Senior Project Director, Center for Health Policy, Columbia University School of Nursing, New York, New York.; **Jingjing Shang, PhD, RN, OCN, FAAN**, is a Professor of Nursing, Center for Health Policy, Columbia University School of Nursing, New York, New York.; **Komal P. Murali, PhD, RN, ACNP-BC**, is an Assistant Professor, NYU Rory Meyers College of Nursing, New York, New York.; **Lori King, MPH**, is a Research Analyst, Center for Home Care Policy & Research, VNS Health, New York, New York.; **Charity Ogunlusi, MBBS, MPH,** is a Graduate Research Assistant, Center for Health Policy, Columbia University School of Nursing, New York, New York.; **Suning Zhao, MPH**, is a Junior Data Analyst, Center for Health Policy, Columbia University School of Nursing, New York, New York.; **Jung A. Kang, PhD, MSN, RN, AGACNP-BC, AGCNS-BC**, is a Graduate Research Assistant, Center for Health Policy, Columbia University School of Nursing, New York, New York.; **Yihong Zhao, PhD, MPhil,** is a Professor of Data Science, Center for Health Policy, Columbia University School of Nursing, New York, New York.; **Khadra Dualeh, MPH**, is a Project Coordinator, Center for Health Policy, Columbia University School of Nursing, New York, New York.; **Margaret V. McDonald, MSW**, is an Associate Vice President, Center for Home Care Policy & Research, VNS Health, New York, New York.

## Abstract

Palliative care improves the quality of life for seriously ill patients, but misconceptions and knowledge gaps hinder its implementation in home healthcare (HHC). This study developed and pilot-tested HHC-specific questionnaires to measure palliative care knowledge, attitudes, and confidence (PC-KAC) among clinicians, patients, and caregivers. Using literature reviews, expert input, and cognitive interviews, the questionnaires were refined to ensure clarity, practical relevance, and content validity. Pilot testing revealed widespread confusion about palliative care, with patients and caregivers often conflating it with hospice care and holding misconceptions about opioid use for pain and symptom management. While clinicians demonstrated adequate knowledge, gaps in pain management and confidence in handling emergencies were evident. These findings highlight the need for targeted education and training to integrate palliative care effectively into HHC, improving patient outcomes and supporting interdisciplinary collaboration.

## Introduction

Palliative care, a patient- and family-centered approach, provides specialized care to individuals with serious illnesses (Kelley & Morrison, 2015). Although traditionally prevalent in hospitals or outpatient settings, there has been a shift toward the preference for home-based palliative care in recent years (Eze, 2023; Roberts et al., 2021). This transition offers significant benefits to patients and their families, including effective management of physical and psychosocial symptoms, reduced healthcare resource utilization, increased documentation of advanced care planning, decreased in-hospital death rates, and improved quality of life and support for caregivers (Diop et al., 2017; Ernecoff et al., 2020; Hyden et al., 2020; Isenberg et al., 2020; Lustbader et al., 2017; Naoki et al., 2018). However, only a small percentage of home healthcare (HHC) agencies currently provide home-based palliative care services, revealing a significant gap between the demand and the availability of specialized care in home-based settings (Cole et al., 2023; Fasolino et al., 2024; Pujolar et al., 2022; Stober, 2017). This gap indicates a significant opportunity to expand palliative care within HHC practice (Eze, 2023).

**Figure:**
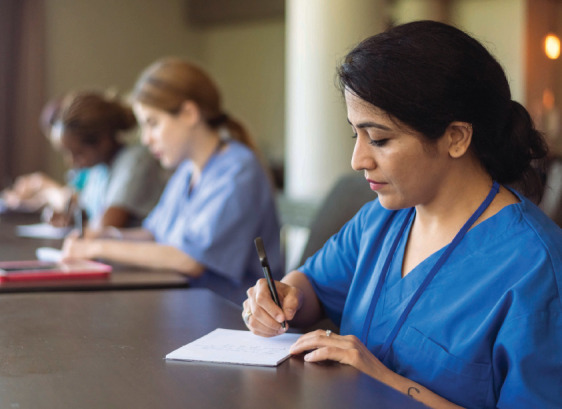
No caption available.

The current shortage of professionals qualified to provide community-based palliative care necessitates proactive initiatives to attract, train, and retain skilled individuals for these roles (Stewart, 2022). Given the potential benefits, the importance of assessing HHC clinician readiness for palliative care integration cannot be overstated. Research has shown that while palliative care training can improve HHC clinicians' knowledge and confidence, a considerable portion still did not feel comfortable having conversations about advanced directives and advanced care planning (Gasper et al., 2018). Therefore, before widescale integration, it is essential to measure HHC clinicians' knowledge, attitudes, and confidence related to palliative care.

Furthermore, understanding patient and caregiver perspectives is crucial for successfully incorporating palliative care into HHC, as limited studies show prevalent misconceptions about palliative care among patients and caregivers, including fears of forfeiting medical treatment (Ankuda et al., 2018; Bowman et al., 2019; Coulourides Kogan et al., 2022). Therefore, gaining a better understanding of the perceptions of palliative care among seriously ill HHC patients and their caregivers can provide valuable insights into improving home-based palliative care programs in HHC and addressing training gaps.

However, comprehensive measures of palliative care tailored specifically to the HHC setting are currently lacking, and no existing survey instruments fully address all eight domains specified by the National Consensus Project's Clinical Practice Guidelines for Quality Palliative Care (Table 1; Ferrell et al., 2018; Murali et al., 2022). Therefore, this study aimed to develop and test two questionnaires assessing palliative care-related knowledge, attitudes, and confidence (PC-KAC) for HHC clinicians, patients, and caregivers.

**Table 1.** National Consensus Project Clinical Practice Guidelines for Quality Palliative Care (NCP Guidelines)

**Domain 1:** Structure and process of care
**Domain 2:** Physical aspects of care
**Domain 3:** Psychological and psychiatric aspects of care
**Domain 4:** Social aspects of care
**Domain 5:** Spiritual, religious, and existential aspects of care
**Domain 6:** Cultural aspects of care
**Domain 7:** Care of patient nearing the end of life
**Domain 8:** Ethical and legal aspects of care

## Methods

All study activities were approved by the Institutional Review Boards at Columbia University Irving Medical Center and VNS Health.

First, we conducted a literature search; reviewed existing instruments, scales, and surveys; and compared them with the eight domains specified in the NCP Guidelines (Ferrell et al., 2018). The applicability of each item to home health care (HHC) was assessed, with questions adjusted to better fit HHC scenarios. New items incorporating HHC-specific content were developed in consultation with a panel of five experts in home-based palliative care and HHC. Through three iterative sessions, the expert panel scrutinized each question for its content validity, readability, comprehension, and ease of response. Feedback gathered from this process was used to inform refinements of the questionnaires.

Ten clinicians (5 registered nurses [RNs], 3 physical therapists, and 2 social workers) and 10 patients/caregivers from a large, urban HHC agency in the Northeast region of the U.S. participated in in-depth cognitive interviews. Participants reviewed the questionnaires, marked items that were confusing or uncomfortable, and noted questions or topics that were missing based on their experiences. During the interviews, the questionnaires were reviewed section by section noting any suggestions, ideas, or questions from participants. Additionally, participants were asked to “think out loud” about specific questions (identified by the study team and experts) using probes to understand comprehension, confidence in the answer, recall, and specificity. Insights from these interviews informed the further refinement of the questionnaires.

Finally, a pilot test was conducted with the final questionnaires to assess test-retest reliability. Thirty-five clinicians (17 RNs, 14 physical or occupational therapists, and 4 social workers), and 15 patients and 16 caregivers from the same large, urban agency in the Northeast participated in the pilot testing. Before completing the questionnaire, verbal consent was obtained from patients and caregivers, while clinicians were provided an information sheet with study information. The questionnaires were administered twice via REDCap electronic data capture tools hosted at Columbia University Irving Medical Center (Harris et al., 2019; Harris et al., 2009), with a 14-day interval between administrations. Clinicians received an email with an anonymous link to complete the questionnaire; in 2 weeks' time, they received an email to complete the questionnaire again. Patient and caregiver questionnaires were administered over the phone or over Zoom with a research assistant inputting answers into RedCAP. Patients and caregivers had the second timepoint scheduled 2 weeks after the first timepoint was completed. For clinicians, the questionnaires took 30-45 minutes (on average) to complete, and 20-35 minutes for patients and caregivers. Thirty-one out of the 35 clinicians and 28 out of 31 patients/caregivers completed the second timepoint.

Due to the non-normal distribution of the raw data, a percentage agreement method was employed to evaluate test-retest reliability (Hartmann, 1977; Saelens et al., 2006; Singh et al., 2011) of the first and second questionnaire timepoints for clinicians, patients, and caregivers. To evaluate the internal consistency, we calculated Cronbach's Alpha for each section of the questionnaires (Cronbach, 1951). Descriptive statistics were computed on participants' first timepoint (*n* = 35 clinicians; *n* = 31 patients/caregivers) using Stata 17 software (StataCorp, College Station, TX), as it indicates their baseline knowledge before being primed to the topic of palliative care.

## Results

### Questionnaire Development

The PC-KAC questionnaires are divided into three main sections: Knowledge (K), Attitudes (A), and Confidence (C), encompassing all eight domains specified by the NCP guidelines. Additionally, both instruments gather participants' prior experiences with palliative care, as well as demographics. In the patient/caregiver questionnaire, their preferences for communication methods and information sources are also collected to provide context and facilitate future targeted interventions. As described in Table 2, the clinician questionnaire is comprised of 101 items, including 86 shared items and additional role-specific items. The patient/caregiver questionnaire contains 71 items, with 65 shared and 6 items specific to either patients or caregivers. Core questions are included for all groups, ensuring comprehensive coverage of palliative care principles. Example questions include statements about provision of palliative care, symptom relief, and management of emergencies.

**Table 2.** Questionnaire Sections and Number of Items

**PC-KAC Clinician Questionnaire**			
**Sections**	**Total**	**Shared**	**Role-Specific**
Knowledge	46	38	8-RN, 2-Therapist
Attitudes	33	33	0
Confidence	16	9	6-RN, 1-Therapist, 1-SW
Preferences/Experiences	6	6	0
**Total**	101	86	
**PC-KAC Patient/Caregiver Questionnaire**			
**Sections**	**Total**	**Shared**	**Role-Specific**
Knowledge	30	30	0
Attitudes	18	17	1-PT
Confidence	9	4	5-CG
Preferences/Experiences	14	14	0
**Total**	71	65	

Note: PC-KAC: Palliative Care-Knowledge Attitude Confidence; RN: Registered Nurse; SW: Social Worker; Therapist: Physical, Occupational, or Speech Therapist; PT: Patient; CG: Caregiver

In the Knowledge section, participants respond to statements by selecting “true” or “false” and answer “all that apply” questions where they can choose multiple applicable options. The Attitudes section contains items where participants indicate their level of agreement with statements about palliative care, using a 5-point scale ranging from “strongly agree” to “strongly disagree.” The Confidence section measures participants' assurance using a 4-point scale from “very confident” to “not confident at all.”

At the beginning of the questionnaire, definitions of serious illness are provided to ensure clarity before participants began the knowledge section (National Institute on Aging [NIA], 2021). These definitions are tailored differently for clinicians and for patients and caregivers to accommodate their varying levels of healthcare-related knowledge. After the definition, participants are prompted with: “Having heard the definition of ‘serious illness’, I will first ask you some questions about your understanding of palliative care in the home.” In the patient/caregiver questionnaire, a short description of palliative care is given after the knowledge section, thereby introducing the concept. This description is a combination of two definitions developed by the Center to Advance Palliative Care and the American Cancer Society (ACS, 2023; CAPC, 2023).

### Cognitive Interviews

During cognitive interviews, specific feedback was gathered about each questionnaire's length, the clarity of questions and topics, and the response options. Overall, participants appreciated the inclusion of response options like “don't know” and “unsure,” which helped improve flow and comprehensibility of the questionnaires.

More specifically, patients and caregivers felt that the definition of palliative care provided in the questionnaire was too academic and recommended adding more practical and relatable examples. One caregiver stated, “I'm not really sure what palliative care is based on definition given — doesn't really come ‘alive’. You should add living examples that are quick to read.” Patients and caregivers also suggested that questions about caregiver appreciation should be considered for inclusion in the questionnaires, and that the differences between palliative care and hospice should be highlighted.

One caregiver also shared a poignant experience, expressing regret over declining palliative care for a loved one due to a lack of understanding of what it entailed. “I wish I knew about what palliative care was. Because when my husband was in the hospital, a doctor told me my husband was eligible for palliative care, but I said no because I didn't know what it was. He was alone in the hospital because I was home bound, and I would talk to him on the phone but it wasn't enough.” While one patient reflected on what broader palliative care service provision might mean for those living with serious illness, “In having conversations about palliative care, you might think you were dying and have a realization of how serious your illness is.” These reflections underscore the critical need for better education and clearer communication about palliative care to ensure patients and families can make informed decisions and receive the support they need.

During cognitive interviews with HHC clinicians, participants found the questions understandable but noted that some might have difficulty recognizing generic pharmaceutical names. They also recommended using terms like “legal primary caregiver” or “Power of Attorney” instead of “surrogate decision maker” for better clarity. These interviews highlighted the need for interdisciplinary collaboration and formal training in palliative care within the HHC setting. An occupational therapist suggested, “It would be wonderful to have disciplines discuss and provide a role in palliative care.” While a social worker emphasized, “It would be helpful for us home healthcare clinicians to have a minimum amount of formal training in palliative and hospice care.”

### Pilot Testing

Participants came from diverse backgrounds in terms of their roles, age, gender, race/ethnicity, education level, and experience working in HHC (limited to clinicians; Table 3). Pilot testing revealed several key insights regarding the knowledge, attitudes, and confidence of HHC clinicians and patients/caregivers related to palliative care. The following results consider participants' first questionnaire responses only.

**Table 3.** Pilot Participant Demographics

Participant Characteristic	Clinicians Total = 35	Patients/Caregivers Total = 31
**Role**	**% (*n*)**	
Field Nurse (RN/LPN/LVN) or Nurse Supervisor	49% (17)	
Therapist (Physical or Occupational)	40% (14)	
Social Worker	11% (4)	
Patient		48% (15)
Caregiver		52% (16)
**Age**		
18-60 years	83% (29)	35% (11)
61 years and older	14% (5)	65% (20)
Prefer not to answer	3% (1)	0% (0)
**Gender**		
Female	86% (30)	81% (25)
Male	11% (4)	19% (6)
Prefer not to answer	3% (1)	0% (0)
**Race**		
American Indian/Alaska Native	3% (1)	6% (2)
Asian/Asian American	29% (10)	0% (0)
Black/African American	17% (6)	32% (10)
White/Caucasian	45% (16)	58% (18)
Other	3% (1)	13% (4)
Prefer not to answer	3% (1)	0% (0)
**Ethnicity**		
Hispanic/Latino	6% (2)	13% (4)
Not Hispanic/Latino	88% (31)	87% (27)
Prefer not to answer	6% (2)	0% (0)
**Education**		
High school diploma and below	0% (0)	25% (8)
Some college or Associates degree	11% (4)	29% (9)
Bachelor's degree	29% (10)	23% (7)
Master's degree and above	57% (20)	23% (7)
Prefer not to answer	3% (1)	0% (0)
**Years Working in Home Healthcare**		
Less than 8 years	17% (6)	
8 or more years	80% (28)	
Prefer not to answer	3% (1)	

Among the 31 patients and caregivers (Table 4), 48% had never heard of palliative care, 16% knew it only by name, 23% had some knowledge, and only a few (13%) had sufficient knowledge of palliative care. When asked about palliative care experiences, only 13% had ever received those services, and 38% were aware of at least one person in their social circle that had received palliative care services.

**Table 4.** Descriptives of Patient/Caregiver Questionnaire Responses∗

**Overall Knowledge of Palliative Care, %(n)**	**Never Heard of it**	**Only Know by Name**	**Some Knowledge**	**Quite a Bit/Very Knowledgeable**
	48% (15)	16% (5)	23% (7)	13% (4)
**Knowledge of Palliative Care, %**	**Correct**	**Incorrect**		
Structural Aspects# (19 items)	68%	32%		
Physical Aspects (4 items)	39%	61%		
Psychological/Psychiatric Aspects (1 item)	100%	0%		
Spiritual/Cultural Aspects (3 items)	72%	28%		
End-of-Life Aspects (1 item)	86%	14%		
Ethical/Legal Aspects (2 items)	66%	34%		
**Attitudes Towards Palliative Care, %**	**Positive**	**Neutral**	**Negative**	**Unsure/Don't Know**
Structural Aspects (5 items)	71%	4%	15%	10%
Physical Aspects (4 items)	32%	9%	36%	23%
Psychological/Psychiatric Aspects (3 items)	85%	1%	4%	10%
Social Aspects (1 item)	100%	0%	0%	0%
Spiritual/Cultural Aspects (2 items)	68%	0%	4%	28%
End-of-Life Aspects (2 items)	81%	5%	6%	8%
Ethical and Legal Aspects (1 item)	97%	3%	0%	0%
**Confidence Regarding Palliative Care, %**	**Very Confident**	**Confident**	**Slightly Confident**	**Not Confident at all**
Overall Confidence (Caregiver only—1 item)	44%	37%	13%	6%
Physical Aspects (12 items)	23%	49%	9%	19%
Psychological/Psychiatric Aspects (2 items)	34%	38%	4%	24%
Spiritual/Cultural Aspects (3 items)	37%	52%	6%	5%
End-of-Life Aspects (2 items)	28%	34%	8%	30%
Ethical and Legal Aspects (Caregiver only—1 item)	40%	60%	0%	0%
**Palliative Care Preferences and Experiences**				
**Preferences, %(*n*)**	**Strongly Agree/Agree**	**Neutral**	**Disagree/Strongly Disagree**	**Unsure/Don't know**
I would consider palliative care for myself or a loved one if they had a serious illness	94% (29)	3% (1)	0% (0)	3% (1)
I think palliative care is best received at home	75% (23)	6% (2)	13% (4)	6% (2)
I want the home healthcare team to ask me about end-of-life planning	48% (15)	0% (0)	42% (13)	10% (3)
**Experiences^, %(*n*)**	**Yes**	**No**	**Unsure/Don't know**	
Ever received palliative care services	13% (2)	81% (13)	6% (2)	
Aware of at least one person from circle of family, friends, or acquaintances that has received any palliative care services	38% (6)	50% (8)	12% (2)	

∗ **Descriptives were calculated exclusively from the first instance of the questionnaire**

# **Participants were skipped out of these questions if their prior palliative care knowledge level was: “never heard of palliative care” or “only heard of it by name,” total skipped *n* = 20**

^ **Participants were skipped out of these questions if their prior palliative care knowledge level was: “never heard of palliative care,” total skipped *n* = 15**

When asked about specific knowledge on symptom management, 65% correctly understood that opioids can relieve severe pain. However, 74% incorrectly believed that opioids are always addictive, and 87% were unaware that morphine can help relieve difficulty in breathing. Overall, the majority (61%) lacked correct knowledge of pain management and opioid use. Regarding spiritual and cultural aspects, 61% correctly identified that spiritual stress can contribute to physical pain, whereas 74% understood that cultural beliefs might change during treatment.

Patients and caregivers also demonstrated notable misconceptions about ethical and legal aspects of palliative care. A majority (58%) incorrectly believed only a family member can make medical decisions for a seriously ill person if they are too sick to do so themselves. Patient and caregiver attitudes regarding palliative care among HHC patients were generally positive. However, attitudes regarding pain management/opioid use varied; most (41%) were negative or did not have enough knowledge (29%) to respond confidently. Despite these gaps, 94% of patients/caregivers either strongly agreed or agreed to consider palliative care for themselves or their loved ones facing a serious illness. Additionally, 75% believe that the best place for palliative care is at home. However, only 48% wished for the HHC team to initiate an end-of-life planning discussion. The preferred sources of information on palliative care for most patients and caregivers were healthcare providers and printed materials.

Among the 35 clinicians (Table 5), 37% had provided hospice and/or palliative services in the past, and 46% had received training in palliative care. When asked about palliative care experiences, only 6% had ever received those services, and 57% were aware of at least one person in their social circle that had received palliative care services. When asked about specific knowledge, 45% incorrectly answered questions about pain management and opioid use, highlighting significant knowledge gaps in this area. Misconceptions included the belief that addiction is a major issue when morphine is used on a long-term basis (69% incorrect) and that opioids commonly cause respiratory depression when used regularly (83% incorrect). Knowledge on end-of-life issues (38% incorrect) and ethical and legal aspects (54% incorrect) was also lacking. Despite these gaps, clinicians overwhelmingly had positive attitudes toward palliative care, with 100% agreeing that patients with serious illness have a right to receive palliative care, and 97% acknowledging the importance of educating HHC clinicians about providing palliative care. However, only approximately 54% of HHC clinicians felt confident in providing palliative care for patients with serious illness in the home.

**Table 5.** Descriptives of Clinician Questionnaire Responses∗#

**Knowledge, %**	**Correct**	**Incorrect**		
Structural Aspects∗ (5 items)	75%	25%		
Physical Aspects (19 items)	65%	35%		
Psychological/Psychiatric Aspects (6 items)	73%	27%		
Social Aspects (2 items)	76%	24%		
Spiritual/Cultural Aspects (4 items)	93%	7%		
End-of-Life (5 item)	62%	38%		
Ethical/Legal Aspects (5 items)	46%	54%		
**Attitudes, %**	**Positive**	**Neutral**	**Negative**	**Unsure/Don't Know**
Structural Aspects (9 items)	90%	4%	5%	1%
Physical Aspects of Care (2 items)	73%	11%	11%	5%
Psychological/Psychiatric Aspects (1 item)	100%	0%	0%	0%
Social Aspects (3 items)	97%	2%	1%	0%
Spiritual/Cultural Aspects (5 items)	92%	5%	2%	1%
End-of-Life (10 items)	74%	11%	12%	3%
Ethical and Legal Aspects (3 items)	74%	10%	15%	1%
**Confidence, %**	**Very Confident**	**Confident**	**Slightly Confident**	**Not Confident at all**
Overall Confidence (1 item)	11%	43%	37%	9%
Structural Aspects (1 item)	3%	20%	37%	40%
Physical Aspects of Care (5 items)	13%	31%	22%	34%
Psychological/Psychiatric Aspects (2 items)	21%	41%	25%	13%
Social Aspects (1 item)	29%	51%	15%	5%
Spiritual/Cultural Aspects (2 items)	17%	49%	22%	12%
End-of-Life (2 items)	18%	37%	28%	17%
Ethical and Legal Aspects (1 item)	24%	44%	16%	16%
**Palliative Care Preferences and Experiences**				
**Preferences, %(*n*)**	**Strongly Agree/Agree**	**Neutral**	**Disagree/Strongly Disagree**	**Unsure/Don't know**
I have easy access to palliative care specialists when I take care of patients with serious illness.	40% (14)	32% (11)	17% (6)	11% (4)
**Experiences, %(*n*)**	**Yes**	**No**	**Unsure/Don't Know**	
Ever received palliative care services	6% (2)	94% (33)	0% (0)	
Aware of at least one person from circle of family, friends, or acquaintances that has received any palliative care services	57% (20)	43% (15)	0% (0)	
Ever worked in hospice or palliative care	37% (13)	63% (22)	0% (0)	
Ever had training in palliative care (webinars, clinical rotation, coursework, certification, etc.)	46% (16)	48% (17)	6% (2)	

∗ Descriptives were calculated exclusively from the first instance of the questionnaire

# Field RN, *n* = 16

When analyzing test-retest reliability (14-day interval), in the patient/caregiver questionnaire (*n* = 30 for two administrations), all three sections demonstrated excellent agreement levels (Saelens et al., 2006) with an average of 84% agreement for the Knowledge section, 79% for Attitudes, and 82% for Confidence. Similarly, in the clinician questionnaire (*n* = 30 for two administrations), all three sections showed robust agreement, with an average of 84% for the Knowledge section, 89% for Attitudes, and 80% for Confidence.

To evaluate the internal consistency, we calculated Cronbach's Alpha for each section of the questionnaires. In the patient/caregiver questionnaire, the results were as follows: 0.89 for the knowledge section, 0.93 for attitudes, and 0.89 for confidence, all indicating high internal consistency. Similarly, the clinician questionnaire showed alphas of 0.91 for the knowledge section, 0.92 for attitudes, and 0.90 for confidence, confirming the reliability of these sections for our research purposes. These results affirm that both questionnaires are robust tools for assessing their respective groups.

## Discussion

Here, we developed and tested the first HHC setting-specific questionnaires measuring palliative care-related knowledge, attitudes, and confidence, providing significant insights into the current state of palliative care readiness and receptiveness among HHC clinicians, patients, and caregivers. Our findings highlighted several key areas where knowledge and confidence gaps persist, emphasizing the need for targeted education and training interventions to improve palliative care delivery in the HHC setting.

Feedback from cognitive interviews with patients and caregivers emphasized the importance of clear delineation between the definitions of palliative care and hospice. Participants pointed out that many families are often confused about the distinctions between these two types of care, which can cause delays in accessing appropriate services. Educational materials that specifically address the goals, timing, and scope of palliative care versus hospice care are needed; distinct, plain-language definitions could aid decision-making by patients and families, ensuring that patients receive the most suitable care based on their individual needs and preferences.

In cognitive interviews with clinicians, it was suggested that the questionnaires include clearer definitions and more practical examples to better capture the nuances of palliative care practices. This would aid in accurately assessing the effectiveness of palliative care interventions and identifying areas for improvement. Additionally, the need for interdisciplinary collaboration and formal training in palliative care was emphasized. Better integration of palliative care principles into the curriculum of healthcare professionals' education and continuous education will ensure that all healthcare providers are adequately prepared to deliver comprehensive and high-quality, patient-centered palliative care.

Results from our pilot test revealed that many HHC patients and caregivers lack adequate knowledge about palliative care. Misconceptions about pain management were widespread and centered around negative attitudes and perceptions about opioid use and addiction. This issue has been observed globally, though studies indicate clinicians, patients, and caregivers in the U.S. generally have better knowledge regarding opioid use in palliative care compared to those in other countries (Makhlouf et al., 2020). Effective palliative care, including appropriate use of opioids, ensures comprehensive management of physical, psychological, and spiritual symptoms, thus improving overall patient outcomes (Marchetti et al., 2013; Weiss et al., 2001). Knowledge gaps around opioid use can hinder effective symptom management, which is essential for seriously ill patients, as it can greatly improve their quality of life by reducing pain and discomfort.

Additionally, misconceptions, like the belief that palliative care is only for end-of-life situations, can affect patients' and caregivers' ability to make informed decisions about care options, while also increasing stress for caregivers who may feel overwhelmed due to unfamiliarity with available resources. Without fully understanding the goal and spectrum of palliative care services, patients and caregivers may be reluctant to utilize these services, resulting in unmet care needs related to their serious illness and consequently, unnecessary hospitalizations and increased healthcare costs (May et al., 2018). The potential impact upon patient outcomes underscores the importance of conveying the benefits of palliative care as an adjunctive model of care within HHC through educational initiatives, such as informational brochures and workshops. Empowering patients and caregivers with knowledge can lead to a more collaborative and supportive care environment in the home, ensuring that their physical, emotional, and spiritual needs are met throughout their healthcare journey.

HHC clinicians, despite demonstrating adequate overall knowledge, had significant knowledge gaps, especially regarding pain management and opioid use. Almost half of the clinicians incorrectly answered questions on these topics. Although this is not a novel finding, it echoes previous research and underscores the need for enhanced training focused on pain management strategies and the safe use of opioids (Makhlouf et al., 2020).

Given their knowledge levels, confidence among clinicians varied, with only a limited number feeling confident in providing palliative care to seriously ill patients in their home. This lack of confidence was particularly evident for managing palliative care emergencies, offering complementary therapies, and conducting end-of-life discussions, suggesting specific areas where further support and education are necessary. Ensuring that HHC clinicians are adequately trained and confident in providing palliative care can significantly enhance the quality of care for patients with serious illnesses. Future training programs should focus on filling the identified knowledge gaps and improving communication skills for navigating difficult conversations, as well as practical, scenario-based learning to build confidence in real-world settings. Strengthening communication skills for end-of-life discussions is particularly important and has already been identified as a key area for improvement (Osakwe et al., 2023). Training on how to conduct non-threatening conversations should also be included, as these conversations are pivotal in aligning care with patient and caregiver preferences.

Despite the knowledge and confidence gaps, we found that attitudes toward palliative care were generally positive among HHC clinicians, patients, and caregivers. All clinicians agreed that patients with serious illness have a right to receive palliative care, and almost all acknowledged the importance of educating HHC clinicians about palliative care. Similarly, almost all patients and caregivers expressed a willingness to consider palliative care for themselves or their loved ones, with most believing that home is the best place for such care. These favorable attitudes, despite the existing knowledge gaps, indicate a readiness among both groups to embrace palliative care services if provided with adequate information and support.

From a healthcare policy perspective, integrating palliative care into the standard care model for HHC could lead to better patient outcomes, reduced hospitalizations, and overall cost savings for the healthcare system. However, our findings highlight the need for training and education around palliative care for HHC clinicians, patients, and caregivers. Policymakers should consider establishing formal palliative care training requirements for HHC clinicians and ensuring that these services are adequately funded and supported. Given the demonstrated willingness of patients and caregivers to engage with palliative care, policies that promote awareness and education about these services are essential.

In the meantime, it is crucial to leverage additional resources to address gaps in introducing patients and caregivers to palliative care services. Many patients and caregivers already have established relationships with their primary care providers (PCPs) and specialists, which can foster a more conducive environment for discussing the benefits of palliative care and facilitating referrals. Relatedly, most of our patient/caregiver survey participants agreed that it is the responsibility of doctors (84%) and nurse practitioners (71%) to inform all patients with serious illness about palliative care. However, while these established relationships between PCPs, specialists, and patients are viewed as uniquely positioned, they do not always lead to palliative care referrals due to misconceptions and challenges with initiating difficult conversations (Coulourides Kogan et al., 2022). Therefore, more research is needed to identify gaps in palliative care knowledge, attitudes, and confidence among referring healthcare providers. Despite these barriers, by strengthening relationships with external healthcare providers, the HHC industry can help ensure that patients and caregivers are properly informed and connected to appropriate palliative care services when needed.

## Conclusions

Pilot testing confirmed the reliability and validity of the PC-KAC questionnaires, which are setting-specific tools for assessing palliative care knowledge, attitudes, and confidence among HHC clinicians, patients, and caregivers. Our findings underscore the need for continuing education and training to address knowledge gaps and improve the confidence of clinicians in providing palliative care, as well as educational opportunities for patients and their caregivers. Moving forward, broader dissemination of the questionnaires will help identify specific training and educational needs nationwide, ultimately laying the groundwork for integrating high-quality palliative care into HHC practice. Future research should focus on adapting these tools for different cultures and countries to enhance the global applicability of the findings and support the widespread implementation of palliative care in the HHC setting.
